# Efficacy Ozone Therapy in Reducing Periodontal Disease

**DOI:** 10.3390/ma16062375

**Published:** 2023-03-16

**Authors:** Giulia Tetè, Teresa D’Amicantonio, Elisabetta Polizzi

**Affiliations:** 1 Vita-Salute San Raffaele University, Dental School Department of Dentistry, IRCCS San Raffaele Hospital, Via Olgettina 48, 20132 Milan, Italy; 2 Center for Oral Hygiene and Prevention, Vita-Salute San Raffaele University, Dental School Department of Dentistry, IRCCS San Raffaele Hospital, Via Olgettina 48, 20132 Milan, Italy

**Keywords:** ozone therapy, oral infection, periodontal disease, non-surgical therapy

## Abstract

The aim of this study is to highlight the properties of ozone as an aid to non-surgical therapy compared to non-surgical therapy alone. This study included thirty Caucasian patients (eighteen women and twelve men) aged between 35 and 65, recruited at the Oral Hygiene and Prevention Center of the Dental Clinic of the Vita-Salute San Raffaele University, at the San Raffaele hospital in Milan. The periodontal probing was recorded with a PC-PUNC 15 manual probe (Hu Friedy) at time 0; the scaling and root planing session was performed at T1 with or without the aid of ozone therapy, and then, the patients were re-evaluated at one month (T2), three months (T3), and six months (T4). The results obtained show that there are not statistically significant differences in terms of reduction in clinical periodontal indices such as plaque, bleeding, and pocket depth between the two groups. Therefore, treatment with ozoral gel would not seem to improve non-surgical periodontal therapy alone. However, clinical periodontal indices significantly improved in patients treated with non-surgical therapy and ozone gel. From this point of view, ozone gel can be used as an aid to non-surgical therapy due to its excellent characteristics, in particular, its powerful virucidal action.

## 1. Introduction

Periodontitis is a multifactorial disease which is born from an alteration of the balance between host, environment, and immune system [1]. The progression of the lesion is certainly mediated by the attack of bacteria that colonise the tooth surface, the gingival margin, and the subgingival environment in a susceptible host [2,3].

At the peak of this pathology, there is the loss of dental elements, which, together with other factors such as the increase in average age and associated comorbidities, has led to a growing need for rehabilitation of edentulous areas and a consequent advancement of minimally invasive procedures [4,5,6,7,8,9,10,11,12].

In the case of implant-supported fixed prostheses, the corresponding phenomenon of periodontitis is peri-implantitis; however, as has been demonstrated by several Authors [13,14,15,16,17,18,19,20], the correct pre- and post-surgical treatment of patients, combined with constant monitoring and inclusion in a periodic professional hygiene maintenance program, could reduce the incidence of this phenomenon and, consequently, of implant failure.

It follows that the patient who presents pathological periodontal clinical indices must be supported by the professional with a precise and accurate diagnosis, an oral hygiene session with motivation and good home hygiene. While the prevention of periodontal diseases is based on microbial agents that inhibit bacterial plaque through two mechanisms, the former provides an inhibition of the primitive plaque, inhibiting bacterial proliferation before division, while the latter has a bactericidal action [21].

Research in recent years has focused precisely on this topic, finding the best antimicrobial to prevent the destruction of periodontal tissues. The literature agrees on the antimicrobial action of chlorhexidine, considered the gold standard of anti-plaque chemical agents, above all for its high substantivity. However, chlorhexidine has side effects such as peeling of the mucous membrane, impaired wound healing, pigmentation, and taste impairment [22].

Some authors point out that several strategies have been employed to accelerate tissue regeneration using bioactive molecules. In recent years, platelet concentrates derived from the patient’s own blood have been used as a regenerative strategy; some authors tested a new liquid platelet formulation prepared without the use of anti-coagulants (injectable platelet-rich fibrin, i-PRF) that was compared to the gold standard, platelet-rich plasma (PRP) with gingival fibroblasts cultured on smooth, rough titanium implant surfaces. Laboratory analyses aimed to observe the proliferation of molecules as well as the expression of platelet-derived growth factor (PDGF), transforming growth factor-β (TGF-β), collagen1 (COL1) and fibronectin (FN). The results show that i-PRF had a highly positive influence on cells in terms of proliferation. Therefore, derivatives without anti-coagulants will assume a key role in translational research [23]. Several studies focus on the need to create biomaterials without anticoagulants because of their side effects [24].

Some authors, on the other hand, have emphasised chitosan as a useful biomaterial in many dental fields including periodontal regeneration, due to its positive effects and its versatility and ease of use [25]. Some authors, on the other hand, have emphasised chitosan as a useful biomaterial in many dental fields including periodontal regeneration, due to its positive effects and its versatility and ease of use, for example, Muzzarelli et al. reported its use in the treatment of 52 periodontal defects with great results due to the molecule’s very architecture [26].

The pandemic caused by the SARS-CoV-2 virus has expanded scientific knowledge around the world. In our sector, it has emerged that good oral health prevents the onset of viral infections. As a result, researchers tested commercially anti-plaque microbial agents for their effectiveness against viruses and particularly against SARS-CoV-2. Several studies have shown that chlorhexidine is not totally effective against viruses; for this reason, ECDC indications and national ministerial guidelines suggest a rinse with chlorhexidine followed by another with hydrogen peroxide before starting the dental procedures [27,28].

A valid alternative to chlorhexidine could be ozone; it is considered a powerful antimicrobial, especially in its ability to counteract viral reduplication by inhibiting the activity of reverse transcriptase, a part of the viral protein synthesis. The application of ozone in dentistry is the result of its chemical–physical properties such as immunostimulant, analgesic, anti-hypoxic, detoxifying, antimicrobial, bioenergetic, and biosynthetic action [29,30,31].

Therefore, given the commitment and attention to safety protocols to counter the spread of the SARS-CoV-2 virus, our study aims to highlight the properties of ozone as an aid to non-surgical therapy compared to non-surgical therapy alone, proposing itself as a valid alternative to other microbial agents [32].

## 2. Materials and Methods

### 2.1. Population and Study Design

This study included thirty Caucasian patients (eighteen women and twelve men) aged between 35 and 65, recruited at the Oral Hygiene and Prevention Center of the Dental Clinic of the Vita-Salute San Raffaele University, at the San Raffaele hospital in Milan and who met the inclusion criteria described below.

The periodontal probing was recorded with a PC-PUNC 15 manual probe (Hu Friedy) at time 0, scaling and root planing session was performed at T1 with or without the aid of ozone therapy and then the patients were re-evaluated at one month (phase T2), three months (phase T3), and six months (phase T4).

Inclusion criteria:

- pathological clinical periodontal indices (BOP ³20% PI ³20% PPD ³4 mm);
- presence of at least 20 teeth;
- absence of periodontal treatment (non-surgical therapy) in the last 12 months.

Exclusion criteria:

- treatment with drugs (antibiotics, anti-inflammatories);
- smokers (if former smokers must have stopped for at least 5 years);
- alcoholics (>150 mL of wine/day, >200 mL of beer/day, any spirits);
- chronic viral infections;
- cardiovascular diseases (heart attack, stroke, arterial hypertension, claudication);
- neurodegenerative diseases;
- diabetes mellitus;
- presumed or certain pregnancy;
- medical conditions or medical history requiring antibiotic prophylaxis prior to periodontal treatment.

### 2.2. Study Procedures

The selected patients received information and instructions on the methods and aims of the study. The selection was made based on the inclusion and exclusion criteria described above and those who agreed to participate had to sign an informed consent.

The study population consisted of patients belonging to the Oral Hygiene and Prevention Center of the Dental Clinic of the Vita-Salute San Raffaele University, at the San Raffaele hospital in Milan.

During T0, and therefore in the first visit, the patient’s personal data, medical history, general state of health, and the use of any drugs were recorded. Furthermore, periodontal clinical indices were detected, i.e., PD > 4 mm, bleeding on probing (BOP), and positive plaque index (PI) (>20%).

The enrolled patients were randomised into two groups: a control group (GC) which included periodontal patients undergoing SRP (scaling and root planing), and a test group (GT), which included periodontal patients who were invited to use ozonated gel (Ozoral) at the end of non-surgical periodontal therapy.

At phase T0:

The two groups were included in the following protocol: a first visit which allowed the two groups to be randomised. Both were asked to complete and sign the anamnesis; then, periodontal probing and clinical parameters were performed (PPD = pocket depth, BOP = bleeding index, PI = plaque index, mobility, and furcation involvement).

At the end of the first investigation, the two groups were randomised.

The PC-PUNC 15 probe (Hu Friedy) was used for the detection of clinical periodontal indices. In addition, an erythrosine-based chromatic plaque detector (mira-2-tohager) was used to detect the plaque index (O’s Leary plaque index).

At phase T1:

All patients (test group and control group) underwent root planing with quadrant scaling (under anaesthesia, if necessary) and both groups received proper home hygiene (IOD) instruction. In addition, mechanical instruments (ultrasound with A, P, PS tips), manual instruments (Gracey courettes 7/8–11/12–13/14), and scalers (204 S) were used for the hygiene session, while rubber pads and nupro paste (Cleanic) were used for the polishing procedure.

At phase T2:

One month after the hygiene session, reassessment for PI-BOP-PPD, presence of mobility and furcation involvement was performed. The PC-PUNC 15 probe (Hu Friedy) was used for the detection of clinical periodontal indices.

At phase T3:

Three months after the hygiene session, the reassessment for PI-BOP-PPD, presence of mobility and furcation involvement was performed. The PC-PUNC 15 probe (Hu Friedy) was used for the detection of clinical periodontal indices.

At phase T4:

Three months after the hygiene session, the reassessment for PI-BOP-PPD, presence of mobility and furcation involvement was performed. The PC-PUNC 15 probe (Hu Friedy) was used for the detection of clinical periodontal indices.

For the control group:

- an oral hygiene session was also performed with supra/subgingival debridement, the patient was motivated with the correct home oral hygiene instructions (IOD) and the recommendation not to use any home antiseptic device after the session.

For the test group:

- An oral hygiene session was performed with supra/subgingival debridement with application of ozonated oil during and at the end of the session, using disposable syringes. The patient was motivated with the correct home oral hygiene instructions (IOD) and the recommendation to use ozone oil for four weeks.

## 3. Results

Our study led to results recorded during re-evaluations at phase T2 (one month from SRP), phase T3 (three months from SRP), and phase T4 (six months from SRP).

**Figure 1:**
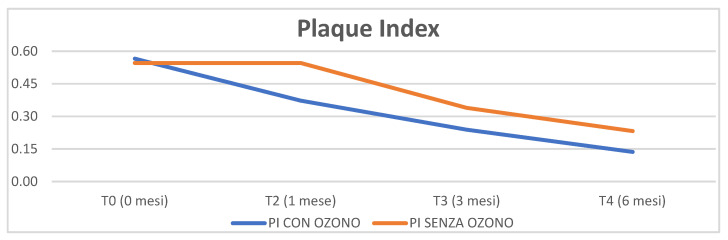
Plaque index parameter analysed through time (0, 1 month, 3 months, 6 months).

The first parameter analysed was the plaque index: clinically there was a decrease over time especially in the test group (with ozone), as can be seen from graph 1. The decrease in the control group (without ozone) was statistically significant *p* = 0.03, while in the test group (with ozone) the decrease tends towards significant *p* = 0.06. However, no statistically significant differences were found between the two groups *p* = 0.62 (Figure 1).

**Figure 2:**
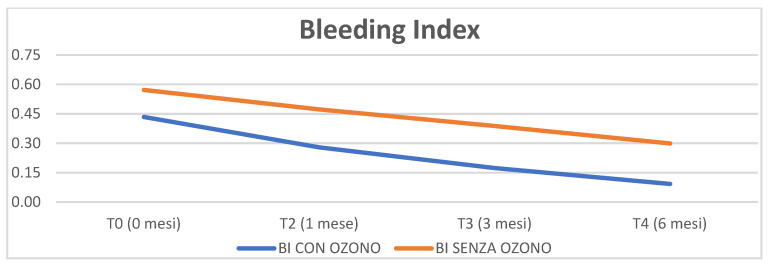
Bleeding index parameter analysed through time (0, 1 month, 3 months, 6 months).

The trend of BI over time (from baseline time to six months) was also considered in our study. Graph 2 shows that there was a reduction in both BI groups, higher in the group treated with ozone. Statistically, the control group had a significant trend *p* = 0.03, while the decrease in the BI of the test group tends to be significant *p* = 0.06. Observing the values of the two groups, from the initial screening to six months, it emerges that the ozone gel has a greater influence in decreasing bleeding than the control group, *p* = 0.05 (Figure 2).

The third parameter analysed is the probing depth (PD). The graph shows that there is a decrease in PD in both groups tending towards significant *p* = 0.06.

**Figure 3:**
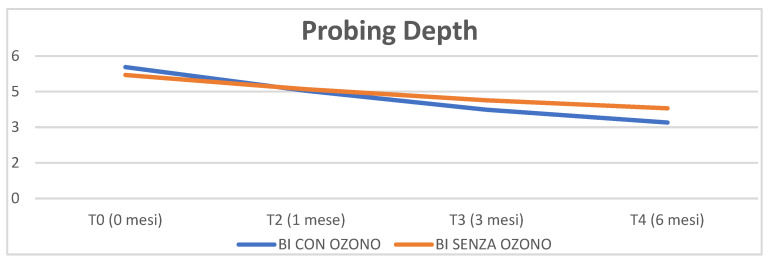
Probing Depth parameter analysed through time (0, 1 month, 3 months, 6 months).

Furthermore, it seems that in the test group, and therefore with the aid of ozone, there is a much higher decrease than in the control but not confirmed by the statistical analysis *p* = 0.07 (Figure 3).

## 4. Discussion

The results obtained show that there are no statistically significant differences in terms of reduction in clinical periodontal indices such as plaque, bleeding, and pocket depth. Between the two groups, therefore, treatment with ozoral gel would not seem to improve non-surgical periodontal therapy alone. However, clinical periodontal indices significantly improved in patients treated with non-surgical therapy and ozone gel. In this regard, the literature underlines that the topical application of ozone gel can improve clinical periodontal parameters. In 2014, Shoukheba saw that the results of ozone irrigation showed improvement of all clinical parameters in the ozone group, which was maintained up to six months, except BOP up to three months [33].

As far as the plaque index is concerned, we did not find significant differences between the test group and the control group, results which confirm the relevant literature; in fact, Tasdemir et al. state that, in a randomised clinical study on 36 patients, there were no statistically significant differences between the two groups treated with and without ozone in terms of plaque indices [34].

Although not statistically significant, an improvement in the bleeding index was found in the ozone-treated test group, a datum confirmed by the literature which indicates improvements in the groups treated with traditional therapy and adjuvants such as ozone therapy and chlorhexidine [35].

Yilmaz achieved statistically significant results in terms of reduction in periodontal clinical indices in patients treated with laser (YAG) and patients treated with topical application of gaseous ozone [36].

Ozone therapy is widely used in medicine and in many branches of dentistry; there is little scientific evidence of its effective use in endodontics and oral surgery. However, it is a growing market and its few risks versus many benefits make it a great aid for non-surgical therapy [37].

Some authors, exploiting the intrinsic characteristics of ozone, have tested it in the disinfection of different materials used for dental impressions. Through an analysis conducted in vitro, the ability to disinfect most commercially available impression materials was tested [38].

In the test group, therefore with the aid of ozone, a much higher decrease was recorded than in the control, but not confirmed by the statistical analysis *p* = 0.07 (Figrue 3) in line with the literature that confirms, for example at two and four months an improvement in pocket depth in the groups treated with traditional therapy and with ozone therapy [39].

In medicine, ozone is widely used for its antimicrobial and antioxidant properties and for biostimulation in the healing of chronic, non-healing or ischemic wounds through various compositions. For topical application, transcutaneous administration of 03 is used if we are talking about external wounds; if we are talking about muscular disorders, through ionised water; and finally, through gels for disorders of the oral cavity. It is also used as an adjuvant to surgical therapy insufflation and or suspension as medicinal oils in the treatment of osteonecrosis of the jaws [40].

To date, a different aspect than clinical efficacy is fundamental. Given the SARS-CoV-2 pandemic, national and international guidelines have been drawn up in the dental world for the safety of patients and operators themselves (our guidelines). Oral hygiene was the dental sector that aroused the greatest concern given the production of aerosols and droplets with the scaler and the use of powders; however, it has been demonstrated that manual therapy, therefore not generating aerosols, achieves the same results as mechanical therapy, through a correct learning curve on the part of the operator [41].

With the advent of the SARS-CoV-2 pandemic, numerous disinfectants have been tested that allow the complete disinfection of surfaces not only from bacteria but also from viruses. Among these, ozone has also been tested. In fact, some authors have proposed an in vitro study in which ozone at different concentrations was tested with the coronavirus family and viral infection decreased by 95% after exposure to ozone for 20 min at 1000 ppmv, 30 min at 100 ppmv and about 40 min at 30 ppmv against the coronavirus family. The results therefore underline that the anti-viral capacity of ozone combined with hydrophilicity favoured a positive surface disinfection result especially on brass, copper and nickel. Overall, this study demonstrates the potential use of ozone gas disinfection to combat the COVID-19 epidemic [42].

In vivo animal studies were conducted to test the toxicity of ozone. Obviously taking into account the limitations of the different anatomy of the first airways, it has been shown that inhalation of ozone causes toxicity mainly at the level of type one cells of the airways, less toxicity to type two cells, and slight biochemical and physiological changes have also been found. However, ozone appears to be a mild mutagen and does not particularly create chromosomal abnormalities. Finally, it can be said from the results obtained that there is a predominantly qualitative but not quantitative difference between species (human–animal), so it can be used to test quantitative toxicity from animals to humans [43].

Other authors point out that the additional use of Xanthan to chlorhexidine gel promoted a greater reduction in PD and an increase in CAL than SRP alone. These results were accompanied by better microbiological and biochemical results when the use of Xan-CHX gel was added to SRP, particularly up to 3 months after treatment [44].

Therefore, in the intermediate period of the pandemic, researchers focused on all possible aids to simple non-surgical mechanical or manual therapy to reduce the risk of aerosol production and still achieve good results. For example, in the literature, chlorhexidine—which is used for the patient before procedures as a rinse, together with povidone iodine, or as an adjunct for non-surgical therapy (ref. chlorhexidine) or laser therapy—shows a significant reduction in clinical indices in patients with periodontal disease [45,46,47].

These aids to normal therapies are not only useful for the SARS-CoV-2 virus but also for all those patients with chronic viral, autoimmune, or cardiac diseases, and therefore, for all patients considered today “fragili” [48].

## 5. Conclusions

Given the results obtained, which consider ozone therapy to be adjuvant to non-surgical periodontal therapy alone; given the conflicting data reported in the literature on the effect of ozone in both surface disinfection and periodontal therapy; and given the limitations of the present study with regard to the limited sample, further in vivo and in vitro studies are needed to test the effect of ozone therapy on periodontal indices, perhaps in the long term.

## Data Availability

Data not available in public databases.
